# Investigation of a novel biofilm model close to the original oral microbiome

**DOI:** 10.1007/s00253-024-13149-8

**Published:** 2024-05-10

**Authors:** Pengpeng Li, Yuwen Zhang, Dongru Chen, Huancai Lin

**Affiliations:** 1 Hospital of Stomatology, Sun Yat-Sen University, Guangzhou, Guangdong China; 2 Guangdong Provincial Key Laboratory of Stomatology, Guangzhou, Guangdong China

**Keywords:** Oral biofilm, 16S rRNA gene sequencing, Oral microbiome, Saliva-derived biofilm, Microbial community

## Abstract

**Abstract:**

A more optimized culture medium used in vitro to mimic the bacterial composition of original oral flora as similar as possible remains difficult at present, and the goal of this study is to develop a novel oral biofilm medium to restore the original oral microbiome. Firstly, we conducted a systematic literature review by searching PubMed and summarized the current reported culture media in vitro*.* Seven culture media were found. We used mixed saliva as the origin of oral species to compare the effects of the above media in culturing oral multispecies biofilms. Results indicated that among the seven media brain heart infusion containing 1% sucrose (BHIs) medium, PG medium, artificial saliva (AS) medium, and SHI medium could obviously gain large oral biofilm in vitro. The nutrients contained in different culture media may be suitable for the growth of different oral bacteria; therefore, we optimized several novel media accordingly. Notably, results of crystal violet staining showed that the biofilm cultured in our modified artificial saliva (MAS) medium had the highest amount of biofilm biomass. 16S rRNA gene sequencing showed that the operational taxonomic units (OTUs) and Shannon index of biofilm cultured in MAS medium were also the highest among all the tested media. More importantly, the 16S rRNA gene sequencing analysis indicated that the biofilm cultured in MAS medium was closer to the original saliva species. Besides, biofilm cultured by MAS was denser and produced more exopolysaccharides. MAS supported stable biofilm formation on different substrata. In conclusion, this study demonstrated a novel MAS medium that could culture oral biofilm in vitro closer to the original oral microbiome, showing a good application prospect.

**Key points:**

*• We compare the effects of different media in culturing oral biofilms*

*• A novel modified artificial saliva (MAS) medium was obtained in our study*

*• The MAS medium could culture biofilm that was closer to oral microbiome*

**Supplementary Information:**

The online version contains supplementary material available at 10.1007/s00253-024-13149-8.

## Introduction

It is evaluated that human mouth has more than 700 different types of bacteria (Aas et al. 2005). The majority of them are related to dental plaque and have formed a highly organized biofilm (Marsh 2005). Microorganisms in biofilm express different genes compared to the planktonic state, resulting in higher resistance to antibiotics, different nutritional needs, or low redox potential, allowing strict anaerobic bacteria to grow under aerobic conditions (Bowden and Hamilton 1998). Biofilm can be thought of as an independent unit rather than a collection of individual bacterial species. Further research should address the biofilm as a whole function (Salli and Ouwehand 2015).

Many oral biofilm cultivation models have been described in previous literature to study bacteria cariogenic mechanisms or caries prevention effects of agents. Most studies use a single-species biofilm to simplify and standardize their models, usually *Streptococcus mutans* (*S. mutans*). However, there is no single species of biofilm in the oral cavity. Even the patient with active dental caries, *Streptococcus mutans* may be a minority species (Aas et al. 2008). Biofilms created from a mixture of several specific bacteria have also been adopted by researchers (Hayati et al. 2011; Rinastiti et al. 2010). However, compared to more than 700 types of oral flora, the biofilm cultured by several specific bacteria can not represent the oral biofilm species as well. How to mimic the complex diversity of oral biofilms cultured in vitro as similar as possible is still difficult at present.

Saliva is often used as the inoculum for oral biofilm culture in vitro. Many studies have tried to culture saliva-derived biofilms to imitate oral microbiomes by using different culture media, but results are quite different. Rudney et al. used basal mucin medium (BMM) to culture saliva-derived multispecies biofilms, and results showed that the average number of positive probes in multispecies biofilms decreased by 1/3 compared to the original saliva (Rudney et al. 2012). Anna et al. used a self-developed SHI medium to culture saliva-derived biofilms, and 454 pyrosequencing results showed that the average number of OTUs detected in vitro cultured biofilms was decreased by more than half of the original saliva samples (Edlund et al. 2013). Kistler et al. used supplementary brain heart infusion (BHI) medium to culture saliva-derived biofilms, and 454 pyrosequencing results showed that the average number of OTUs detected in multispecies biofilms decreased by 1/4 (Kistler et al. 2015). Therefore, there is still a big gap between the microbial community cultured in vitro biofilm model and the original oral biofilm microbiome.

Some key substances, such as heme, vitamin K, and sheep blood, have been reported to be important for the growth of specific bacterial groups. Heme and vitamin K are essential for melanin-producing bacteria species growing, such as *Porphyromonas gingivalis* (Mayrand and Holt 1988). Sheep blood could promote the culture of captious and slowly growing obligate anaerobes in the oral microbiota (Bradshaw et al. 1994). However, the above traditional oral bacterial culture media are usually lack of the key substances. In order to develop a more optimized culture medium which could cultivate multispecies biofilm imitating oral original microbiome, we firstly conducted a systematic literature review by searching one single database PubMed and summarized the currently reported oral multispecies culture medium in vitro (the selected media including BHI, SHI, BMM, and so on). And then, we compared the above culture media in biofilm formation, flora distribution, and species diversity. Thereafter, we added some key supplements to the media in order to obtain a novel medium which would be more suitable for the culture of oral multispecies biofilm imitating oral original microbiome. The mixed saliva was used as the origin of species to compare the effects of the above models in culturing oral multispecies biofilms; crystal violet staining and 16S rRNA gene sequencing were used to compare the amount of biofilm and the distribution of flora. Species diversity was compared between the original oral saliva and biofilms cultured in different media. This study aimed to obtain a more optimized oral biofilm culture medium in vitro, which would contribute to the study of oral biofilm-related diseases in vitro.

## Materials and methods

### Saliva and dental plaque collection

The saliva samples were collected from six healthy adults. Subjects were asked not to receive any systemic disease treatment within 3 months and not to take any prescription or over-the-counter drugs. Before donating saliva, subjects could not eat or drink anything for 2 h. The saliva samples would be combined and centrifuged for 10 min at 2600 g. A Tris–EDTA buffer solution was used to collect dental plaque from the surface of lower molars of the same subjects. Dental plaque (Pla) and saliva (Sa) are stored at − 80 °C. This research was approved by the Ethics Committee of the Hospital Stomatology, Sun Yat-sen University (approval no. KQEC-2021–71-01).

### Cultivating saliva-derived oral biofilms using different media

The 200 µL mixed saliva was added to a 24-well plate. To dry the saliva coating, the plate would be opened at 37 °C for 1 h. Then, the plate was disinfected for 1 h under ultraviolet light. Mixed saliva (10 µL) was added to the pre-coated wells containing 1 mL each of the following media: BHI containing 1% sucrose (BHIs), SHI (Tian et al. 2010), PG (Walther et al. 2019), AS (Li et al. 2019), BMM, tryptone soy broth (TSB), and Roswell Park Memorial Institute 1640 (RPMI) medium. The formula of each medium was shown in supplementary file (Table S1). Biofilms were cultured at 37 °C under anaerobic conditions (85% nitrogen, 5% carbon dioxide, 10% hydrogen) for 24 h.

MAS medium was modified from artificial saliva medium, and we improved it by adding heme, vitamin K, sheep blood, et al. The composition was as follows: 10% fetal bovine serum, 85% artificial saliva, 5% sheep blood, Vit K 1 mg/L, sucrose 10 g/L, haemin 5 mg/L, and arginine 0.174 g/L.

### Crystal violet (CV) staining assay

After cultivation for 24 h, the medium was discarded. Then, the biofilm was rinsed with phosphate buffer saline (PBS) three times, fixed with methanol for 15 min, and dried for 15 min. 0.1% crystal violet dye for 15 min, abandoned crystal violet dye solution, rinsed twice, 300 µL 95% ethanol was added and shaken for 15 min, ethanol was sucked into the blank hole, and the amount of biofilm was evaluated by 600-nm optical density using a microplate reader.

### Analysis of oral biofilm and exopolysaccharide (EPS) by confocal laser scanning microscopy (CLSM)

Oral biofilms were cultured in confocal dishes using BHIs, PG, MAS, and SHI media according to the above methods, and the biofilms cultured for 24 h were rinsed with PBS three times. The LIVE/DEAD BacLight™ Bacterial Viability staining kit (Invitrogen) was used to label biofilms. Then, the biofilm was analyzed by CLSM.

The amount of EPS in biofilms was examined by CLSM. At the beginning of biofilm formation, the dextran conjugate which was labeled by Alexa Fluor 647 was used to label the EPS (Klein et al. 2009; Yang et al. 2016). The biofilm cultured for 24 h was rinsed three times with PBS. Then, the biofilm was stained with SYTO9 green fluorescent dye (molecular probe) for 15 min. Alexa Fluor 647 was detected at 655–668 nm and SYTO9 at 480–500 nm. The images were collected by CLSM. Then, COMSTAT was used to analyze the images (Liu et al. 2017).

### Scanning electron microscopy (SEM) of oral biofilm

Cell-attached slides were placed in a 24-well plate, and the oral biofilm was cultured according to the above method. The above-prepared samples were rinsed gently with an appropriate amount of PBS; dried with absorbent paper; fixed with 2.5% glutaraldehyde; placed in a refrigerator at 4 °C overnight; rinsed with PBS; dehydrated with 30%, 50%, 70%, and 90% series concentrations of ethanol at 4 °C for 15 min; and dehydrated twice with 100% ethanol for 15 min each. The biofilm was observed by SEM after freeze-drying and gold spraying.

### The 16S rRNA gene sequencing

The genomic DNA was extracted from cultured oral biofilms in vitro, dental plaque and mixed saliva, respectively, using the QIAamp DNA kit. The purity and concentration of DNA were detected by agarose gel electrophoresis. The 16S rRNA gene fragment was amplified by PCR using 515F-907R barcoded primers. The fragment was about 500 bp, covering the V4-V5 hypervariable region. The QIAquick PCR purification kit was used to purify PCR amplicons. The library was constructed using the TruSeq ® DNA PCR-Free Sample Preparation Kit. The constructed library was quantified by Qubit and Q-PCR. NovaSeq6000 was used for sequencing.

Bioinformatics analysis was performed using the QIIME program. The sequence was clustered into operational taxonomic units (OTUs) by default with 97% consistency using UPARSE algorithm. The taxonomy was analyzed by Ribosomal Database Project Classifier (min. confidence 0.8) against the Human Oral Microbiome Database. The I-Sanger cloud platform was used to perform the analysis.

### Effects of saliva or dental plaque as inoculum on oral biofilms

The dental plaque samples were collected (Jiang et al. 2020). The saliva coating was obtained in the 24-well plate according to the above method. The 10 µL mixed saliva and dental plaque samples were inoculated into the plate containing 1 mL MAS medium, respectively, and then incubated anaerobically at 37 °C for 24 h. Then, oral biofilms were collected for 16S rRNA gene sequencing according to the above method.

### Effect of different substrata on oral biofilms

Saliva-derived oral biofilms were cultured using MAS medium for 24 h. Hydroxyapatite (HA) slice and glass slice were placed at the bottom of 24-well plates. Then, we collected the biofilms for 16S rRNA gene sequencing.

### The cultivation of single-species biofilm

*Streptococcus mutans* UA159 (ATCC 700610, *S. mutans*) and *Streptococcus sanguis* (*S. sanguis*) ATCC10556 were cultured anaerobically in BHI overnight. *Candida albicans* (*C. albicans*) SC5314 (ATCC MYA‐2876) was cultured in Sabouraud’s dextrose broth at 37 °C and shocked at 200 rpm for 18 h. The cultures were inoculated into MAS, BHIs, PG, and SHI to form biofilms. Then, the amount of biofilm was calculated by crystal violet staining.

### Statistical analysis

Results were analyzed by OriginLab Origin 2022b and SPSS 25.0. All the studies were repeated three times. A significant level was set as *p* value < 0.05.

## Results

### Comparison of 16S rRNA gene sequencing of saliva and supragingival plaque

**Fig. 1:**
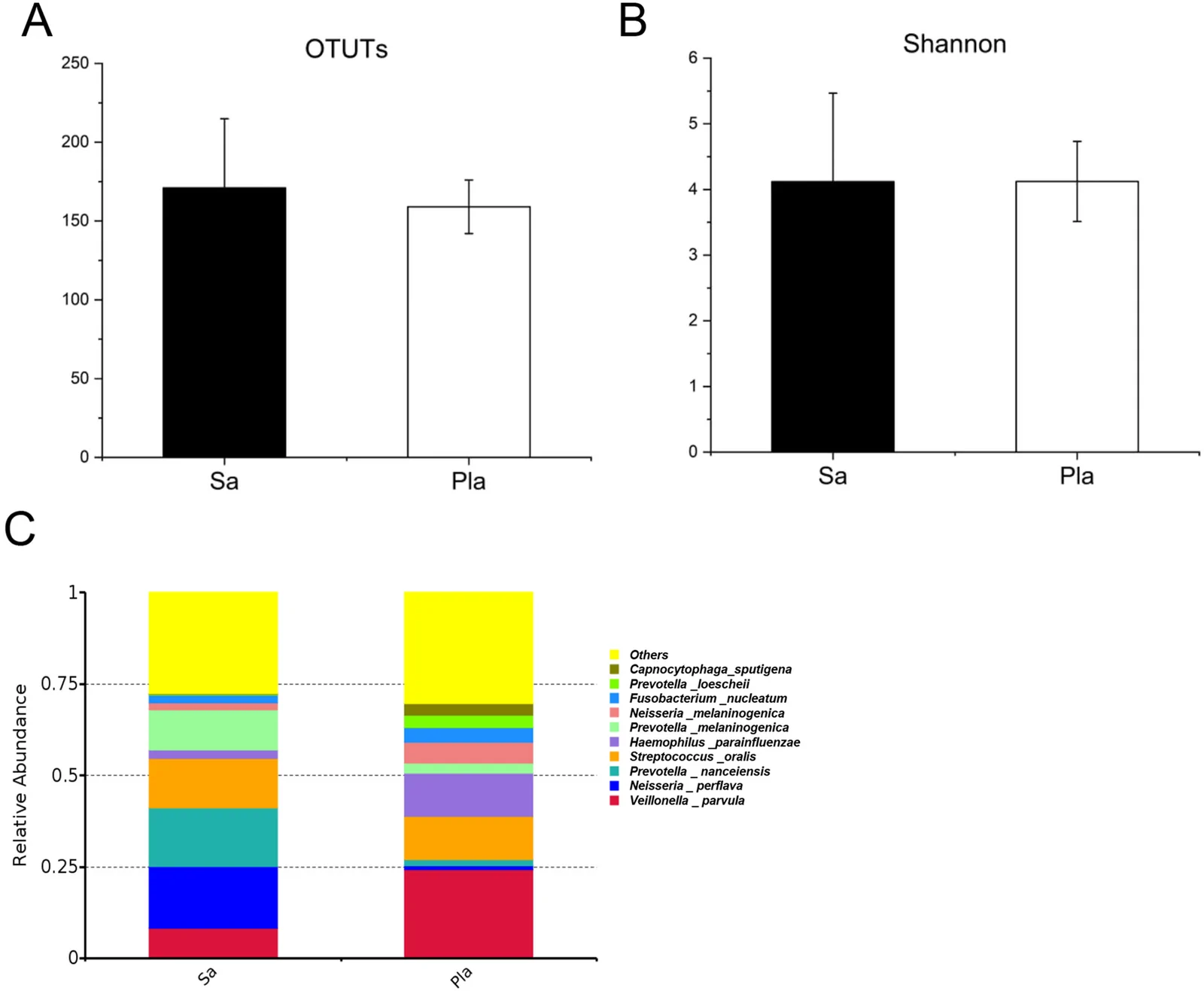
Comparison of the 16S rRNA gene sequencing results of mixed saliva and dental plaque. **A** The OTUs of plaque were smaller than that of saliva (*p* > 0.05). **B** Shannon index of saliva and plaque was similar. **C** The relative abundance of species at genus level Top10 in mixed saliva and dental plaque were presented, respectively

According to the 16S rRNA gene sequencing results, 171 OTUs were obtained from mixed saliva and 159 OTUs from dental plaque. The Shannon index and OTUs were not significantly different between saliva and dental plaque (Fig. 1A and B). At the genus level, the top 10 species with the highest abundance of saliva and dental plaque were basically the same, but the species abundances were different. *Prevotella nanceiensis*, *Neisseria perflava*, *Streptococcus oralis*, and *Prevotella melaninogenica* were higher in saliva, while *Veillonella parvula*, *Streptococcus oralis*, and *Haemophilus parainfluenzae* were higher in dental plaque (Fig. 1C). The rarefaction curves of each sample were shown in supplementary file (Fig. S1 A).

### Effects of the screened media for oral multispecies biofilm cultivation

**Fig. 2:**
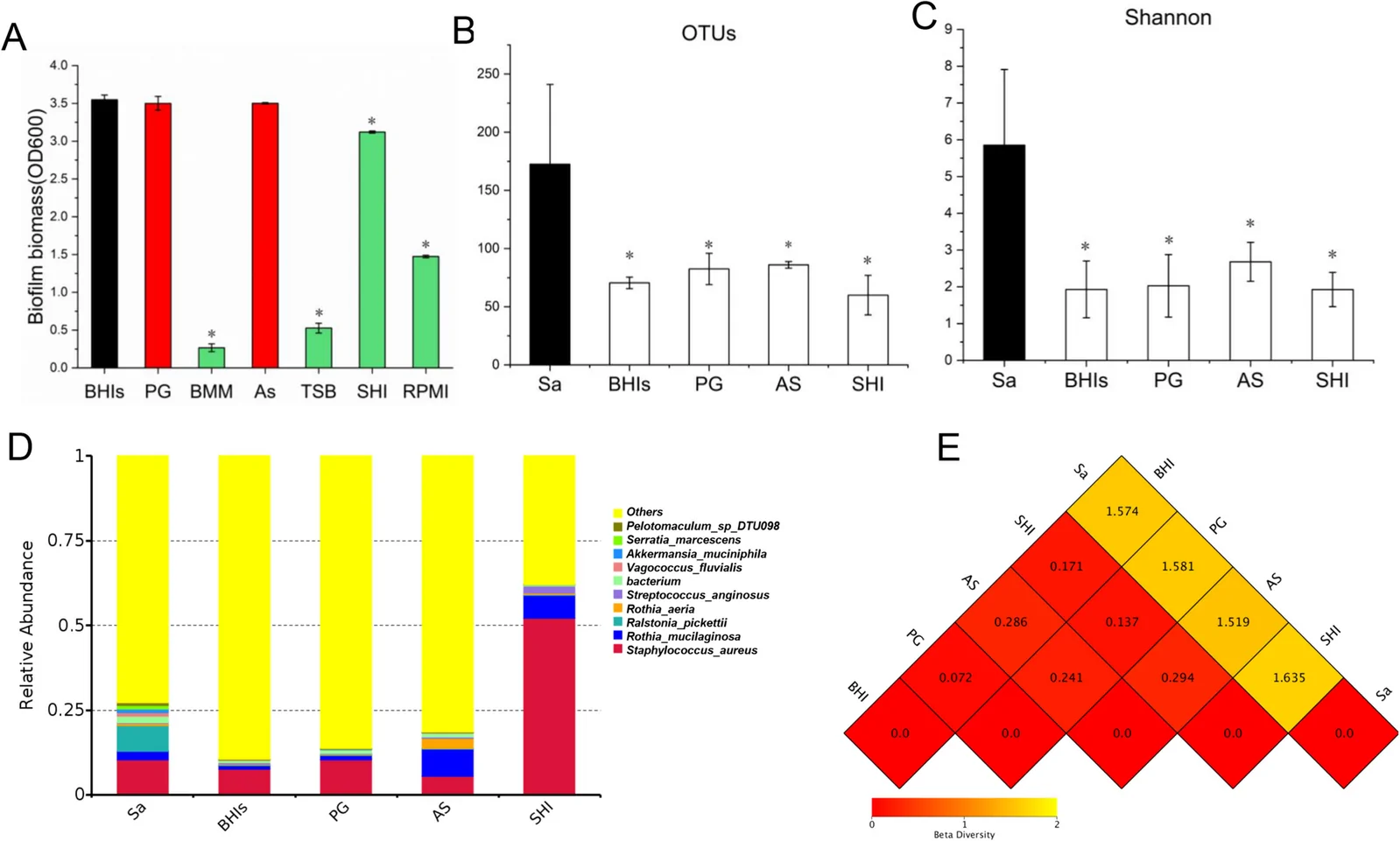
The saliva-derived biofilms cultured in different reported media. **A** The biofilms were cultured in seven different media. The CV staining results showed that biofilm biomass cultured in BHIs, PG, AS, and SHI had large amount (*p < 0.05). **B**, **C** Comparison of the OTUs and Shannon index between the saliva-derived biofilms cultured in different media (BHIs, PG, AS, and SHI) and saliva. Results indicated that OTUs and Shannon index were all greatly decreased in biofilms cultured by different media (*p < 0.05). **D** The relative abundance of species at genus level Top10 showed that there were great differences between the cultured species and original saliva species. The biofilm cultured in AS medium was closest to the relative abundance of saliva species. **E** The distance matrix heat map showed that the dissimilarity coefficient between the AS group and saliva sample was the smallest, indicating that the two samples had the smallest difference in species diversity

The CV staining results showed that oral multispecies biofilms cultured in the above different media were quite different. The commonly used medium BHIs were set as the control group, and results indicated that biofilms cultured by BHIs, SHI, AS, and PG media were in large biomass, while biofilms cultured by BMM, TSB, and RPMI had significantly less biomass (Fig. 2A). Thus, the above four media of BHIs, SHI, AS, and PG were selected for the next experiments.

The 16S rRNA gene sequencing analysis results showed that mixed saliva obtained 170 OTUs, and saliva-derived biofilms grown in the above four media could only obtain 60 ~ 90 OTUs (Fig. 2B). The species abundances described by Shannon index also greatly decreased compared to saliva (Fig. 2C), indicating that a large percentage of oral species could not be cultured in vitro biofilm. Besides, the species abundances at the genus level were also quite different among the four media. As shown, the OTUs and Shannon index of biofilm cultured in AS medium were the highest compared to the other three media, and the relative abundance of species at the genus level also showed that the biofilm cultured in AS medium was closest to the relative abundance of the original saliva species (Fig. 2D). The distance matrix heat map showed that the dissimilarity coefficient between AS group and saliva was the smallest, indicating that the two samples had the smallest difference in species diversity (Fig. 2E). The rarefaction curves of each sample were shown in supplementary file (Fig S1 B).

### Modified media and their effects

**Fig. 3:**
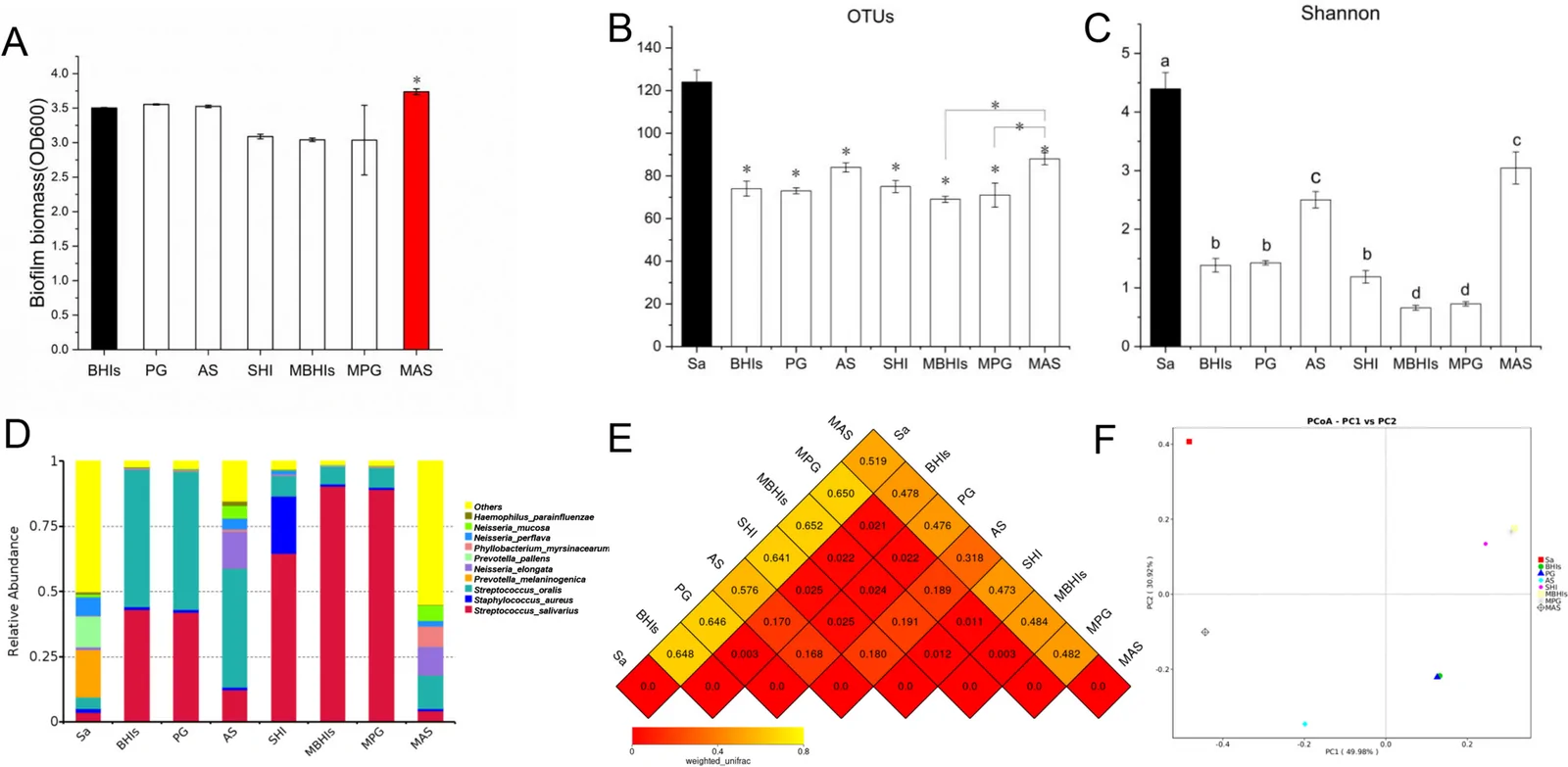
Comparison of the effects of modified media. **A** Three modified media (MBHIs, MPG, and MAS) were compared with the previously reported four media. CV results indicated that the modified medium MAS could achieve the highest biofilm biomass, and the difference was significant (*p < 0.05). **B**, **C** Comparison of the OTUs and Shannon index of biofilms cultured in different media. The modified MAS medium had the highest OTUs and Shannon index compared to other media, but still lower than the original saliva (different superscript letters for different values denote statistically significant differences, *p < 0.05). **D** The relative abundance of species at genus level Top10 indicated that the modified MAS medium was closer to the original saliva species. **E** The distance matrix heat map showed that the dissimilarity coefficient between the MAS group and saliva sample was the smallest, indicating that the two samples had the smallest difference in species diversity. **F** The principal coordinate analysis (PCoA) plot showed that the distance between the MAS group and original saliva sample was the smallest

The above four media caused varying degrees of the loss of microbial species in the original oral microbiota, possibly due to the lack of certain substances in the media, which may be crucial for the growth of oral microorganisms with specific nutritional needs. But we found that a more uniform microbial community was established in the AS medium. We improved the media by adding certain critical supplements, such as heme, vitamin K, and sheep blood, or changing the proportion of original components. Finally, we screened and obtained three modified media (MBHIs, MPG, and MAS) for further experiments. Results of CV staining showed that the biofilm cultured in our modified MAS medium had the highest biofilm biomass (Fig. 3A). As a result of 16S rRNA gene sequencing, the OTUs and Shannon index of biofilm cultured in MAS medium were the highest among all the media (Fig. 3B and C). The relative abundance of species at the genus level also showed that the biofilm cultured in MAS medium was greatly closest to the relative abundance of the original saliva species (Fig. 3D).

In the study of Beta diversity, the distance matrix heat map showed that the dissimilarity coefficient between the biofilm of MAS medium and the original saliva sample was the smallest, indicating that the two samples had the smallest difference in species diversity (Fig. 3E). The principal coordinate analysis (PCoA) plot reflected the degree of difference; the distance between points indicated how different the samples were. The distance between the MAS group and original saliva sample was also the smallest (Fig. 3F). In the PCoA plot, samples that are more similar in their community composition are closer together. The rarefaction curves of each sample were shown in supplementary file (Fig S1 C).

### Biofilm biomass and EPS amount analyzed by CLSM

**Fig. 4:**
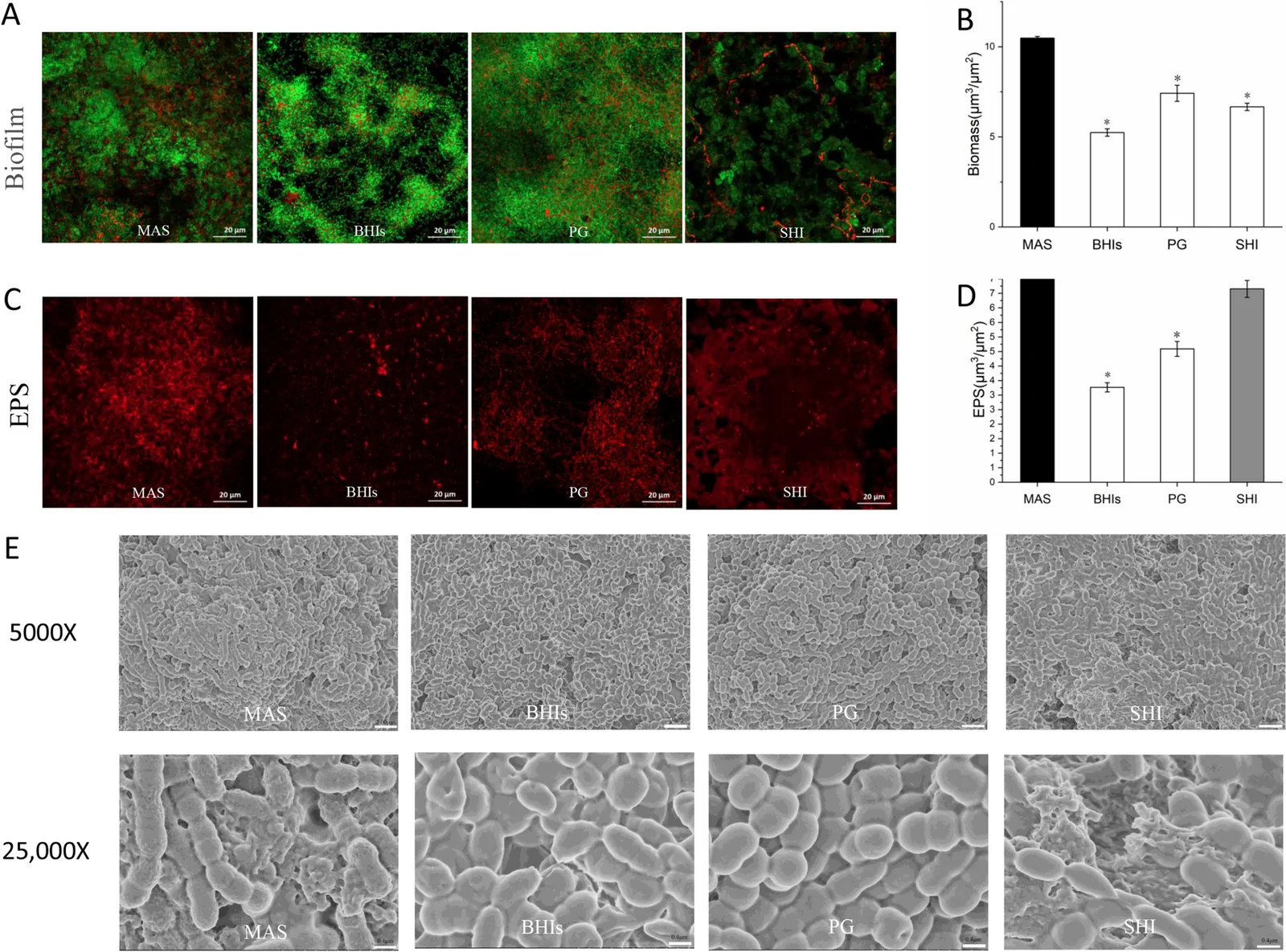
CLSM analysis of oral biofilm and EPS cultured in different media. **A** The live/dead staining indicated that all four media could culture denser biofilms. **B** The biofilm biomass calculated by the three-dimensional reconstruction of CLSM images showed that MAS had the highest biofilm biomass (*p* < 0.05). The MAS group was set as the control. **C** The EPS staining was more obvious in MAS and SHI groups. **D** The EPS amount calculated by the three-dimensional reconstruction of CLSM images also verified that the EPS was higher in the MAS and SHI groups (*p < 0.05). **E** Comparison of the biofilm morphology cultured by different media. Images of biofilms were captured using SEM at × 5000 and × 25,000 magnifications

In our following studies, MAS instead of AS was taken to compare with the other three media of BHIs, PG, and SHI. CLSM images of live and dead species staining showed that biofilms cultured in MAS medium were denser than the other media (Fig. 4A), and the biofilm biomass calculated by the three-dimensional reconstruction of CLSM images also showed that MAS had the highest biofilm biomass (*p* < 0.01, Fig. 4B). For EPS, it showed that the red fluorescence intensity of EPS released in the MAS group was the most obvious as seen in CLSM images (Fig. 4C), and the corresponding statistical results also verified that the amount of EPS in the MAS group was significantly higher than BHIs and PG groups, but the differences between MAS and SHI groups were not significant (*p* > 0.05, Fig. 4D).

### Morphologies of the cultured oral multispecies biofilm observed by SEM

The biofilms cultured by BHIs, PG, SHI, and MAS medium could all form dense biofilms that exhibited complex multilayer structures as observed by SEM at low magnification (Fig. 4E). It could also be seen that the biofilm cultured by MAS was denser than other groups, and the biofilm formed more complex multilayer structures. As observed at the high magnification, a large number of spherical and rod-shaped bacteria connected to each other in a dense accumulation could be seen. In addition, a reticular extracellular matrix was present in the biofilm, which was more obvious in MAS and SHI groups (Fig. 4E). This was consistent with the results observed under CLSM.

### Effects of oral biofilm cultured on different substrata by MAS

**Fig. 5:**
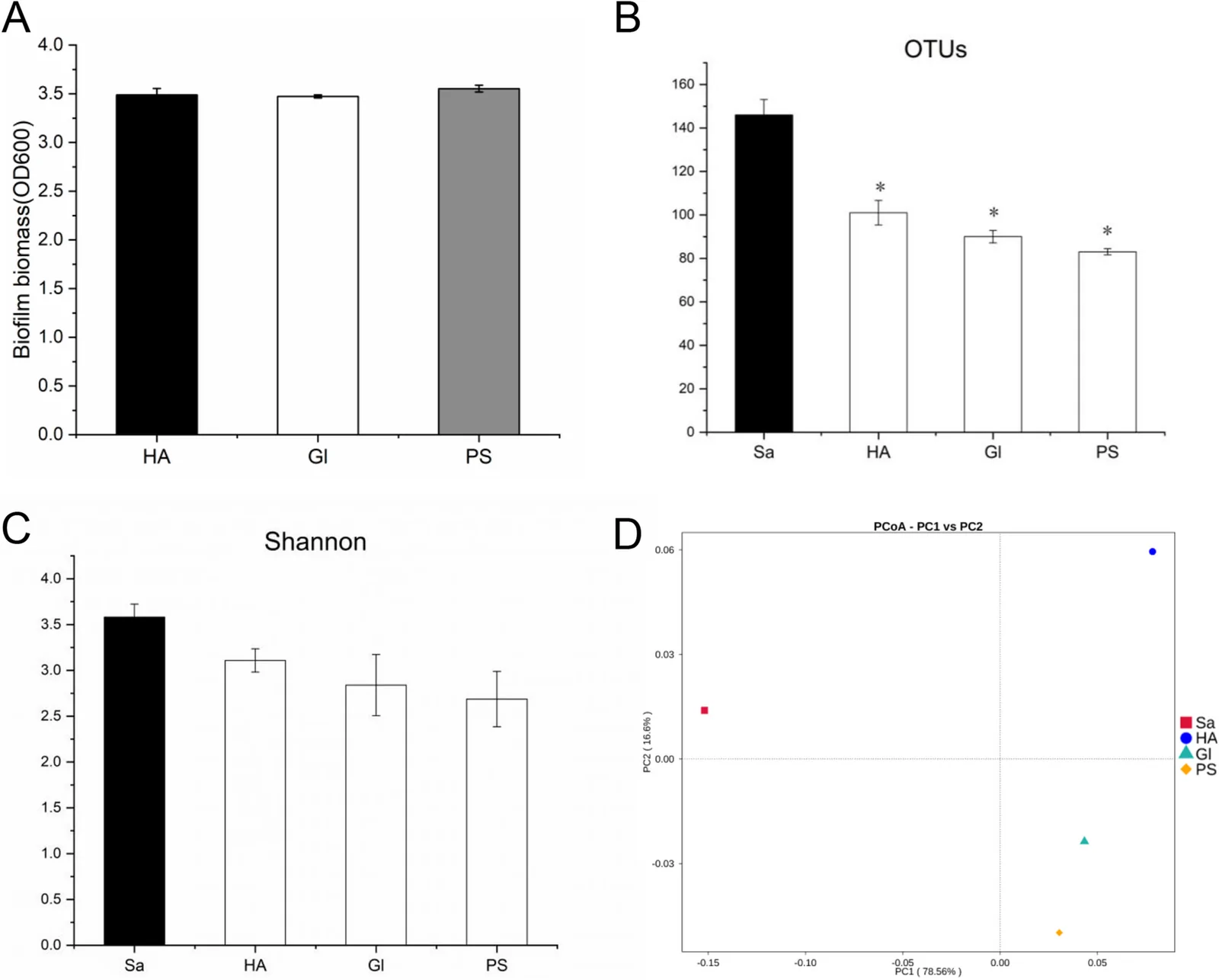
Comparison of effects of oral biofilm cultured on different substrata by MAS. **A** CV staining showed that the biofilm biomass cultured at different substrates (HA, glass, and polystyrene) had no significant differences. The **B** OTUs and **C** Shannon index of biofilms cultured in different substrata also showed similar results. **D** The principal coordinate analysis (PCoA) plot showed the differences among the biofilms cultured at different substrata, indicating the species at the genus levels cultured by MAS were influenced by substrata

To learn the effects of MAS for the cultivation of oral biofilm on different substrata, three commonly used substrata were selected, namely HA, glass (Gl), and polystyrene (PS). Results of CV staining showed that the biofilm biomass cultured on different substrata had no significant difference (Fig. 5A). And 16S RNA gene sequencing results showed that a range of 85 ~ 100 OTUs was obtained from saliva-derived biofilms cultured with different substrata. There was no significant difference in OTUs and Shannon index of biofilms cultured on HA, glass, and polystyrene surfaces (Fig. 5B and C). In Beta diversity studies, the PCoA plot showed that biofilms cultured on HA, glass, and polystyrene surfaces were different (Fig. 5D). It indicated that MAS could support oral multispecies biofilm formation by different substrata, but the species at the genus levels were influenced by substrata. The rarefaction curves of each sample were shown in supplementary file (Fig S1 D).

### Effects of MAS and other media for single-species biofilm cultivation

**Fig. 6:**
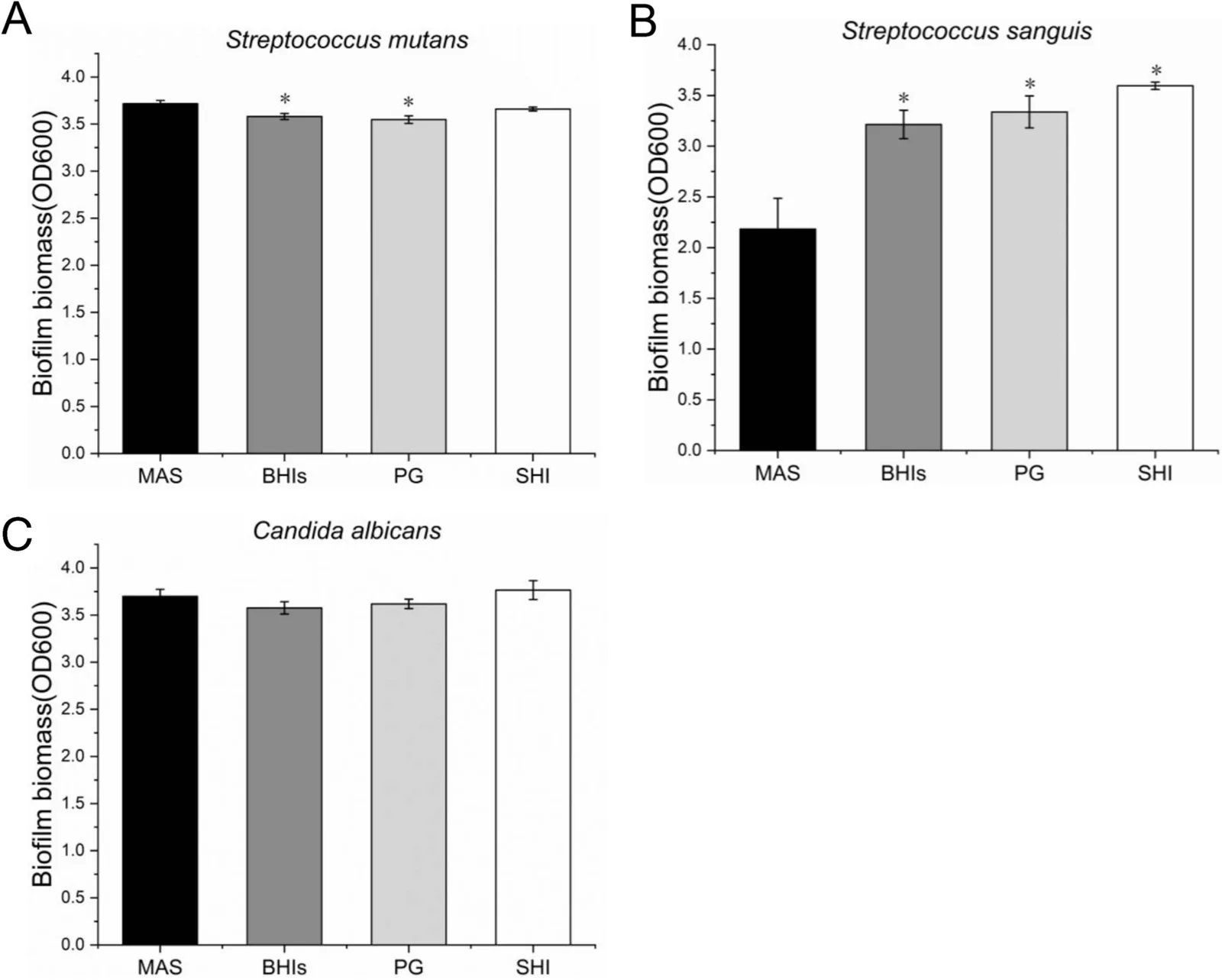
Comparison of MAS and other media (BHIs, PG, and SHI) for single-species biofilm cultivation. **A** All four media could achieve large biofilm biomass of *Streptococcus mutans*, but the MAS group had higher biomass than BHIs and PG groups (*p* < 0.05). **B** The *Streptococcus sanguis* cultured in MAS had the lowest biofilm biomass (*p* < 0.05). **C** The *Candida albicans* cultured in different media had similar biofilm biomass

In order to further explore the effects of MAS on single-species biofilm culture, we selected three microorganisms related to caries, namely *S. mutans*, *S. sanguis*, and *C. albicans*. As a result of CV staining, the biofilm of *S. mutans* cultured in MAS had higher biomass than BHIs and PG, but no significant difference with SHI (Fig. 6A). The biofilm of *S. sanguis* cultured in MAS had the lowest biomass (Fig. 6B). As for *C. albicans*, there is no significant difference between MAS and the other three media (Fig. 6C). It indicated that MAS selectively supported the growth of different oral bacteria.

## Discussion

The development of novel medium to culture oral multiple-species biofilm has been a difficult problem (Kolenbrander 2000). The community structure of saliva-derived biofilm does not exactly match the community structure of dental plaque, although saliva-derived biofilm has been found in most genera with relatively high abundance of dental plaque in vitro. The reason may be that in the plaque ecosystem, fluid flows to provide substances, which is an open culture or batch feeding system. However, oral biofilms are often grown in enclosed systems; oral microbes are supplied with limited nutrients in closed containers (McBain 2009). Besides, some species may be lost because the medium does not fully meet their nutritional needs.

Different medium components may be suitable for different dominant strains. The addition of some small amounts of critical supplements will affect the cultivation effect of biofilm to some extent. BHI medium had been successfully used to culture various picky and non-picky oral bacteria (Moore et al. 1982). Although BHI is a medium of abundant nutrition, it omits materials, such as heme and vitamin K. When mucin, heme, and vitamin K were supplemented, BHI medium could support a highly various bacteria communities (Salli and Ouwehand 2015). However, even BHI with supplementation led to low activity and low species abundance of biofilm in another study (Baraniya et al. 2020). The SHI medium developed by Tian and his colleagues could support diverse oral microbiota (Li et al. 2017; Tian et al. 2010). The added pig stomach mucin had been proven to be the main source of carbon and energy, supporting the growth of oral bacterial communities (Wyss 1989); N-acetyl muramic acid had been proven to promote the culture of some anaerobic subgingival bacterial, like *Tannerella forsythia* (Wyss 1989). In addition, AS medium is also widely used to cultivate complex biofilms, which contain certain proteins, amino acids, various ions, and low molecular weight compounds that may be crucial for the growth of oral microorganisms with specific nutritional needs (Darrene and Cecile 2016).

In order to obtain a more optimized culture method, the reported oral biofilm culture systems were incorporated as much as possible in this study. We optimized the ratio of components and detected their culture effects through crystal violet staining experiments and 16S rRNA gene sequencing analysis and obtained MAS medium finally. As a result of 16S rRNA gene sequencing, MAS medium was better than other media. The newly modified MAS medium could maintain a high degree of diversity of the bacterial community in vitro, which is most similar to the oral bacterial community of the original saliva. However, we also found that *Prevotella*, *Veillonella*, *Amnipila*, *Fusobacterium*, and others tend to disappear or decrease in the MAS culture system compared to saliva. Besides, MAS supported stable biofilm formation on different substrata. In addition, our experimental results confirmed that there were no significant differences in oral biofilms cultured on different non-biological substrata, which was consistent with previously researches (Valappil et al. 2012, 2007); however, Li et al. found that reconstructed human gingiva (RHG), a biological matrix, could support highly active and diverse microbiome (Li et al. 2021).

Oral biofilm models in vitro can be roughly divided into two categories: closed batch culture models and open continuous culture models. The biofilm of a closed batch culture model grows on the bottom of the plate or on solid samples placed in pores, such as hydroxyapatite, resin, or titanium discs. Unlike the real environment in the oral cavity, these models do not have the flow of liquids and nutrients (Chevalier et al. 2018). However, the batch culture model does provide a method for comparing multiple test compounds or conditions simultaneously; they require only a small amount of reagents and are easy to use, reproducible, and economical (Coenye and Nelis 2010). Continuous cultivation includes artificial oral models and flow cell models. They provide intermittent or continuous nutrient flow on biofilms, mimicking in vivo conditions as much as possible (Periasamy and Kolenbrander 2010; Valappil et al. 2014). Due to the complexity of equipment compared to batch culture systems, they typically have fewer replicates. This study is based on a static system, which makes it difficult to simulate the oral saliva environment. Therefore, further research on dynamic in vitro culture systems may be significant.

In this experiment, saliva or plaque samples were collected from multiple donors. Because the taxonomy of saliva samples of different individuals is quite different, if we only study the saliva of one person, some of these oral microorganisms are likely to be missed (Human Microbiome Project 2012). However, the limitation of using several human saliva samples as inoculants in a biofilm model in vitro might be that individual oral microbial integrity is lost (Cieplik et al. 2019). Most likely, everyone involved in this research has a characteristic salivary microbiome. In addition, oral biofilms derived from different batches of the same mixed saliva have been shown to be repeatable (Edlund et al. 2013; Kistler et al. 2015). Saliva is most commonly used as inoculum in vitro oral biofilm models. As our experiments showed, saliva inoculum was not completely similar to dental plaque inoculum acquired from the same donors. We found that every type of inoculant produced a bacterial biofilm reflecting these differences. Saliva is convenient to obtain and easy to collect and disinfect. This study mainly used saliva as inoculum.

The main challenges in most subfields of microbiology remain related to the microbial culture barrier (Vartoukian et al. 2010). It is very desirable to develop an in vitro oral biofilm model with highly diverse bacteria to support the growth of uncultured oral bacteria because it lets us manipulate and study the oral biofilm in a controlled environment (Marsh and Moter 2011). In conclusion, this study demonstrated that the novel MAS medium can culture multispecies biofilm that is closer to the original oral microbiome, and the results are stable, showing a good application prospect.

## Supplementary Information

Below is the link to the electronic supplementary material.

Supplementary file1 (PDF 192 KB)

## Data Availability

The datasets generated and/or analyzed during the current study are available from the corresponding author on reasonable request. The 16S rRNA sequencing raw data was stored in NCBI Sequence Read Archive database: PRJNA937654.
