# Supplementary material for: Investigation of a novel biofilm model close to the original oral microbiome
Source: Appl Microbiol Biotechnol. 2024 May 10;108:330. doi: 10.1007/s00253-024-13149-8 (PMC11087337; doi:10.1007/s00253-024-13149-8)
Supplement: Supplementary file 1 [file 253_2024_13149_MOESM1_ESM.pdf]

Investigation of a novel biofilm model close to the original oral microbiome

Pengpeng Li[1,2], Yuwen Zhang[1,2], Dongru Chen [1,2,*], Huancai Lin [1,2,*]

1 Hospital of Stomatology, Sun Yat-sen University, Guangzhou, Guangdong, China

2 Guangdong Provincial Key Laboratory of Stomatology, Guangzhou, Guangdong, China

*Corresponding author: Dongru Chen, email: chendru@mail2.sysu.edu.cn; Huancai Lin, email: linhc@mail.sysu.edu.cn

AS	artificial saliva solution supplemented with 1% sucrose and 10% fetal bovine serum
MAS	10 % fetal bovine serum, 85 % artificial saliva, 5 % sheep blood, sucrose 10 g / L; hemin 5 mg / L; VitK 1 mg / L; arginine 0.174 g/L
SHI	proteose peptone 10 g/L; trypticase peptone 5.0 g/L; yeast extract 5.0 g/L; KCl 2.5 g/L; sucrose 5 g/L; hemin 5 mg/L; VitK 1 mg/L; urea 0.06 g /L, arginine 0.174 g/L; mucin (type III, porcine, gastric) 2.5 g/L; sheep blood 5% and N -acetylmuramic acid (NAM) 10 mg/L
PG	36 g/L BHI powder, 5 g/L yeast extract, 10 g/L sucrose, 0.4 g/L L-cysteine hydrochloride, 5 mg/L hemin, vitamin K 1 mg/L
BHIs	BHI supplemented with 1% sucrose
MPG	36 g/L BHI powder, 5 g/L yeast extract, 10 g/L sucrose, 0.4 g/L L-cysteine hydrochloride, 5 mg/L hemin, vitamin K 1 mg/L, arginine 0.174 g/L, 10 % fetal bovine serum
MBHIs	37 g/L BHI powder, 10 g/L sucrose,5 mg/L hemin, vitamin K 1 mg/L, arginine 0.174 g/L, 10 % fetal bovine serum
RPMI;TSB; BMM;BHI	commercial media

Table S1. The formula of different culture media.

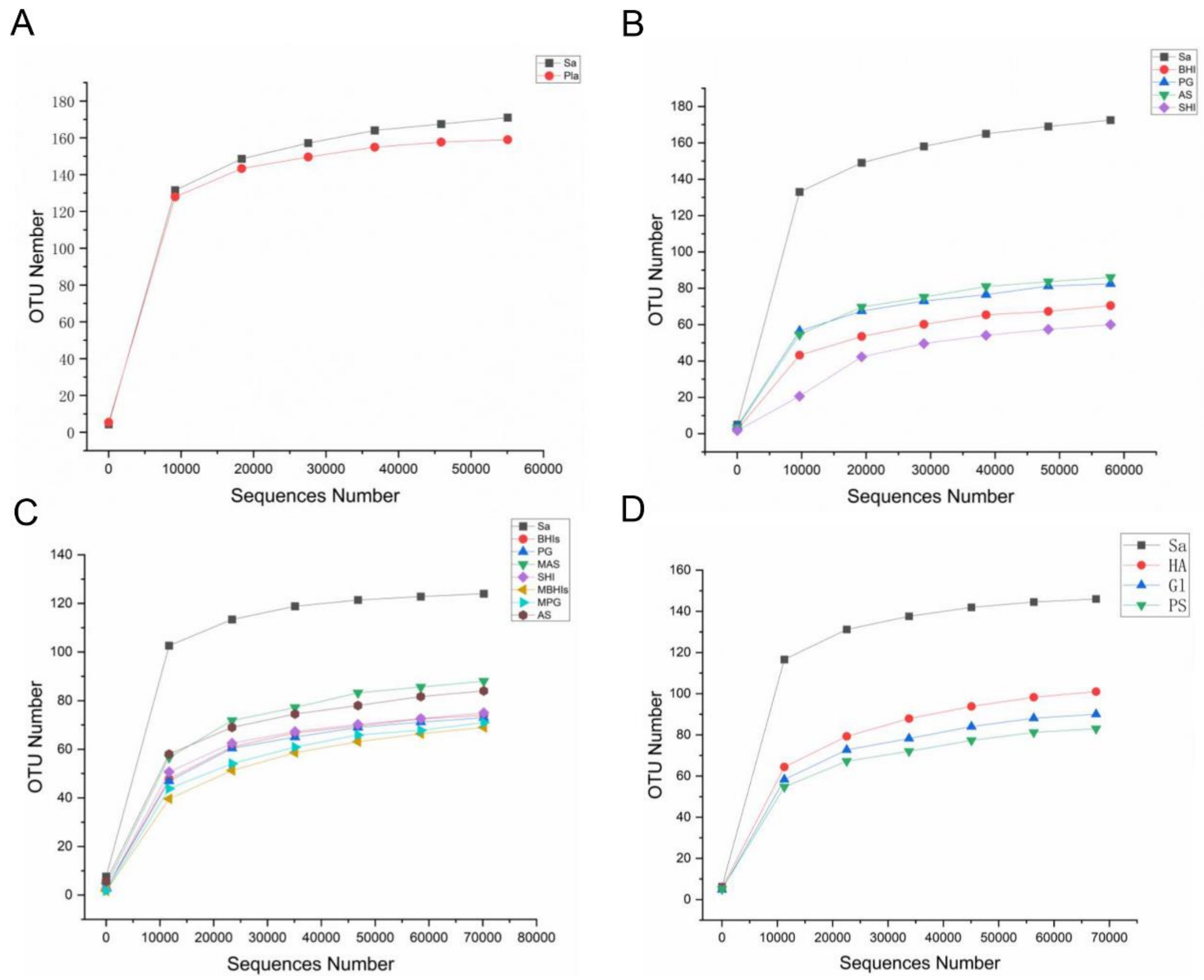

Figure S1. A: Rarefaction curves of each sample in Figure 1 of the main body. B: Rarefaction curves of each sample in Figure 2 of the main body. C: Rarefaction curves of each sample in Figure 3 of the main body. D: Rarefaction curves of each sample in Figure 5 of the main body.
